# Pinpointing Synaptic Loss Caused by Alzheimer’s Disease with fMRI

**DOI:** 10.3233/BEN-2009-0240

**Published:** 2009-10-21

**Authors:** Adam M. Brickman, Scott A. Small, Adam Fleisher

**Affiliations:** ^1^Department of NeurologyTaub Institute for Research on Alzheimer's Disease and the Aging BrainCollege of Physicians and SurgeonsColumbia UniversityNew YorkNYUSA; ^2^Department of NeurosciencesUniversity of CaliforniaSan DiegoCAUSA; ^3^Banner Alzheimer's InstitutePhoenixAZUSA

## Abstract

During its earliest stage, before cell loss and independent of amyloid plaques and neurofibrillary tangles, Alzheimer's disease (AD) causes synaptic loss affecting the basal functional properties of neurons. In principle, synaptic loss can be detected by measuring AD-induced changes in basal function, or by measuring stimulus-evoked responses on top of basal changes. Functional magnetic resonance imaging (fMRI) is sensitive to both basal changes and evoked-responses, and there are therefore two experimental approaches in which fMRI can be used to pinpoint synaptic loss in AD. In this review, we will compare and contrast both approaches for pinpointing when and where synaptic loss in AD begins and for monitoring therapeutic efficacy.

## 1. Introduction

As with all neurodegenerative processes, Alzheimer's disease progresses over time. Anatomically, a range of longitudinal imaging and neuropsychological studies suggest that AD begins in the hippocampal formation [1], presenting with mild forgetfulness [2], before sweeping throughout the neocortical mantle leaving dementia in its wake. Human and animal-model studies suggest that within an affected region, AD progresses pathophysiologically as well [3,4]. Ultimately, AD is characterized by profound neuronal cell death and the deposition of extracellular amyloid plaques and intracellular neurofibrillary tangles. During its earliest pathophysiological stage, however, AD appears to cause 'synaptic failure' before cell death [5], and independent of fully formed plaques and tangles [4]. Pinpointing when and where synaptic failure begins in the hippocampal formation, a structure made of molecularly-distinct subregions, is therefore one of the main promises of fMRI. If this goal can be accomplished, fMRI can be used to: A) Determine where in the hippocampal formation AD begins. This anatomical information can be used as an important first step to understanding underlying mechanisms [6]. B) Detect and perhaps even diagnose AD during its earliest stages. Because synapses are 'plastic', this early stage of the disease provides a time-window during which the disease is most amenable to therapeutic interventions. C) Monitor the efficacy of therapeutic interventions over time.

In clarifying the exact mechanisms that underlie AD-induced synaptic failure, it is instructive to consider the two fundamentally distinct mechanisms by which synaptic function can change in the normal brain: Either by changing the actual number or density of synaptic elements (dendritic spines or presynaptic boutons), through a process called 'structural' plasticity; or, by changing synaptic physiology, without changing synaptic structure, for example by changing stimulus evoked neurotransmitter release or post-synaptic receptor sensitivity. Post-mortem studies in human patients and, more recently, post-mortem studies in animal models of the disease have established that AD-induced synaptic failure is characterized by changes in

ISSN 0953-4180/09/$17.00 © 2009 – IOS Press and the authors. All rights reserved

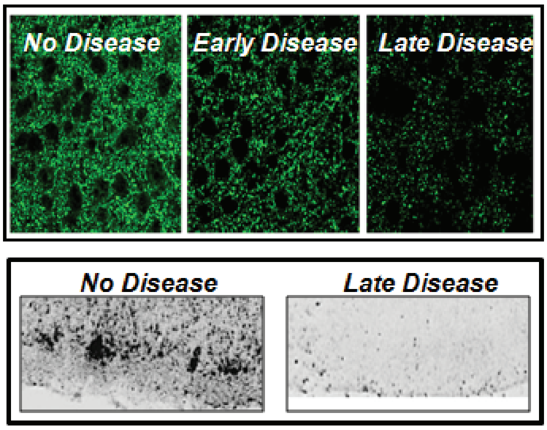

Fig. 1. Mechanism of 'synaptic failure' in Alzheimer's disease. During its earliest synaptic 'failure' stage, Alzheimer's disease is characterized by a loss in the number of synapses, as detected by synaptophysin staining (upper panel; courtesy of Eliezer Masliah), and a reduction in basal metabolism, as detected by cytochrome oxidase staining (lower panel).

synaptic structure – the loss in the number and density of synaptic elements [3,4].

Because it affects the basal properties of neurons, synaptic loss does not require a stimulus evoked response for detection. Synaptic loss can be detected even in post-mortem brains by using, for example, synaptic markers to simply count the number of synapses (Fig. 1A), or by using cytochrome oxidase staining as an indicator of basal oxygen metabolism (Fig. 1B). Nevertheless, synaptic loss will cause a decrease in the amplitude of stimulus induced synaptic responses. Thus, evoked-responses can also detect synaptic loss, and often this approach is superior because it can amplify a relatively subtle changes in synaptic number.

Functional magnetic resonance imaging is, in principle, sensitive to both forms of synaptic plasticity – changes in synaptic 'structure' or changes in synaptic 'physiology' (Fig. 2). As an indirect measure of synaptic function, fMRI relies on the fact that any synaptic changes will affect 'cerebral metabolic rate of oxygen consumption' ($CMRO_2$). Unable to directly measure $CMRO_2$, fMRI measures hemodynamic correlates of metabolism instead – cerebral blood flow (CBF), cerebral blood volume (CBV), or deoxyhemoglobin content (dHB) [7].

Until recently, mapping acute changes in metabolism in response to external sensory stimulation has been the most common use of fMRI (Fig. 2), and in 1991 CBV was the first hemodynamic variable used to map sensory stimulation [8]. However, when in 1992 dHB was shown to also map visual stimulation [9], this approach (called BOLD, for 'blood oxygen level dependent) [10] quickly displaced CBV mapping, mainly because the latter required the injection of IV contrast agents. Subsequent studies have shown that techniques sensitive to shifts in CBF can also be used for mapping sensory stimulation [11].

This brief review of the pathophysiology underlying AD and the neurovascular physiology underlying fMRI explains why there are essentially 2 experimental paradigms that can be used in an effort to use fMRI to pinpoint synaptic loss within the hippocampal formation. The first is to map AD-induced changes in basal metabolism as a reflection of synaptic number (Fig. 2). The second is to use an 'acute-on-chronic' approach, in which external sensory stimulation is used to map evoked-responses on top of the basal changes (Fig. 2).

Here, we will compare and contrast both approaches and opine on which might be best suited for mapping synaptic loss in AD. Because sensory-evoked fMRI is most commonly used, we will begin by reviewing the 'acute-on-chronic' approach, highlighting its strengths and potential limitations. We will then turn to basal approach and its application to AD.

## 2. The 'acute-on-chronic' approach

Even though, as discussed, AD causes a primary change in synaptic number, it is possible to use an 'acute-on-chronic' approach with fMRI in AD. Namely, it is possible to map sensory-evoked fMRI responses, on top of the AD-induced changes in synaptic number (Fig. 2). This approach has many advantages. First, because AD has a very slow time-course, on practical grounds it is challenging to map basal changes over time. So, most imaging studies cross-sectionally compare patients versus controls at a single time-point. As hemodynamic factors are only indirect correlates of function, when cross-sectional differences are observed there is always a concern as to whether they truly reflect neuronal dysfunction. Showing disease-associated differences when using sensory stimulation is therefore a powerful approach for linking imaged changes with neuronal dysfunction. Second, akin to a stress-test in echocardiography, the use of sensory stimulation can enhance the ability to detect neuronal dysfunction, by 'bringing-out-the-lesion'. Third, sensory stimulation is obviously required if the question is how a diseased brain abnormally processes sensory stimulation.

Indeed, beginning in 1999 [12], many studies have used the acute-on-chronic approach to identify differences in the BOLD response when comparing AD patients to healthy controls [13–15].

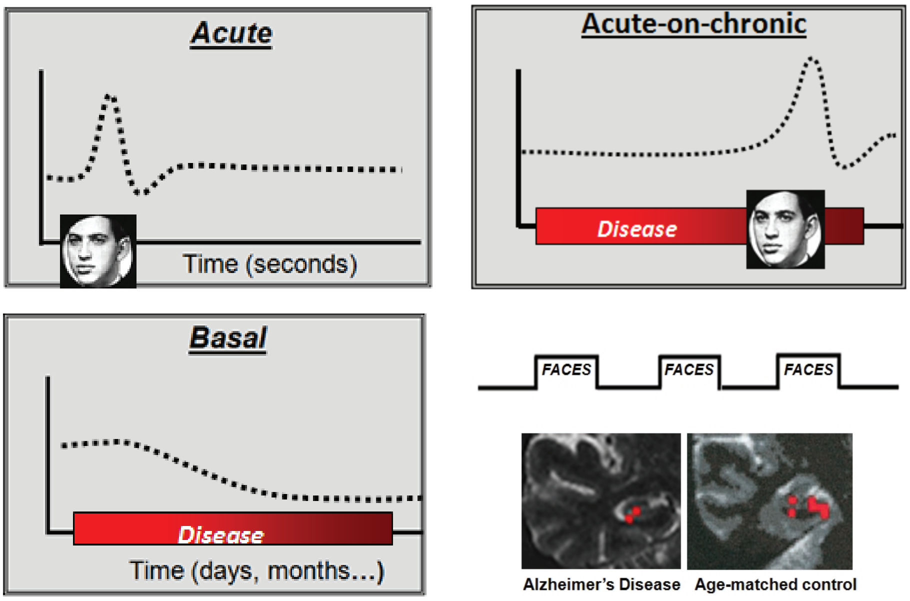

Fig. 2. fMRI approaches for mapping synaptic loss. fMRI can map acute stimulus-evoked changes in metabolism (upper left panel, in which flashing faces illustrate external stimulation), or can map disease-associated changes in basal metabolism (lower left panel). Synaptic loss in Alzheimer' disease can be detected by mapping basal changes or by using an 'acute-on-chronic' paradigm, in which stimulus-evoked changes are measured 'on top' of basal changes (upper right panel). The acute-on-chronic approach has been used to show differences in stimulus-evoked fMRI responses in the hippocampal formation, comparing Alzheimer' disease to healthy controls (lower right panel).

## 3. Potential Limitations of the acute-on-chronic approach

The hippocampal formation is made up of separate but interconnected subregions [16]: the entorhinal cortex, the dentate gyrus, the CA1 and CA3 subfields, and the subiculum. Each hippocampal subregions houses a unique molecular expression profile [17], accounting for why each hippocampal subregion is differentially vulnerable to mechanisms of disease [18]. Thus, when any disease is found to cause hippocampal dysfunction, for example by cognitive testing, it is important to determine which subregion is differentially affected.

For the goal of pinpointing in which hippocampal subregion synaptic loss begins in AD, acute-on-chronic BOLD has 4 limitations (Fig. 3). First, the use of an external stimulus is a potential experimental bias. For example, in one study comparing the BOLD response across different hippocampal subregions, the entorhinal cortex of AD patients was found to show activity evoked by novel faces [12]. Because viewing novel faces might differentially stimulate the entorhinal cortex, it is difficult to conclude that the entorhinal cortex is necessarily differentially affected by AD compared to other hippocampal subregions. Compromised spatial resolution is the second limitation of the acute-on-chronic paradigm. High temporal resolution is required to capture the transient changes in synaptic activity induced by sensory stimulation, and this, by necessity limits spatial resolution. Although it should be pointed out due to recent advances in acquisition and analytic technology, spatial resolution continues to increase [19].

The third limitation is based on a range of recent observations suggesting that the amplitude of the BOLD response can be confounded by basal neurovascular factors that are affected by disease (Fig. 4). One of these factors is termed 'n', where $n = \%\Delta CBF / \%\Delta CMRO_2$ [20]. 'n' is the basic relationship that underlies fMRI across a broad range of functional stimuli, establishing the relationship between changes in CBF and changes in $CMRO_2$. During acute changes in $CMRO_2$, 'n' has been found to vary significantly from region to region [21,22] and in disease state [23], so that stimuli that induce identical changes in underlying function will lead to very different changes in CBF, varying the response recorded by fMRI. Another confounding basal neurovascular factor is termed 'M' [24]. Reflecting the levels of dHB in the basal state, 'M' varies from region to region and is affected by disease [23,25]. As first demonstrated by Davis and colleagues [24] and con-

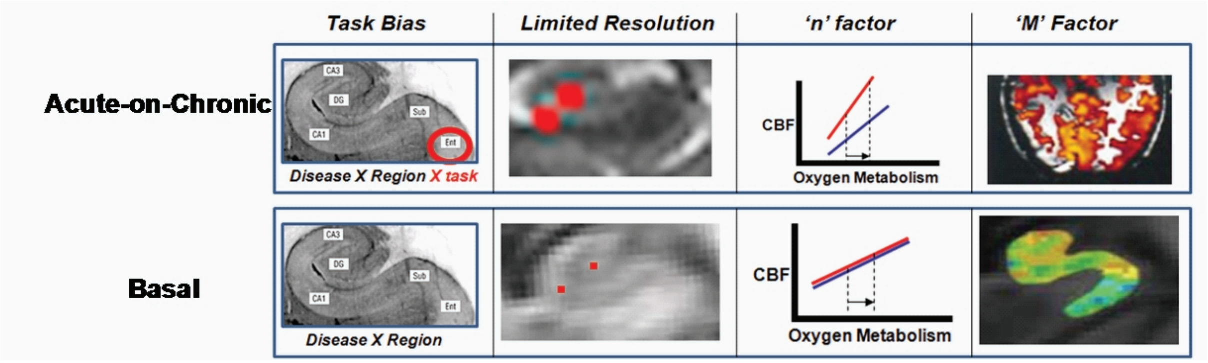

Fig. 3. Comparing acute-on-chronic versus basal imaging. The limitations of the acute-on-chronic approach are illustrated in the top panel, while the bottom panel illustrates how these limitations are partially addressed in the basal approach. **Task bias:** In the acute-on-chronic approach a given 'activation' task might differentially engage a particular hippocampal subregion (the entorhinal cortex in this example, indicated by red circle), which would bias the results to show a between-group difference in this particular subregion, even if this subregion is not differentially targeted by disease. In the basal approach there is no activation task, so observed between-group differences are driven by the disease itself. In statistical terms, the acute-on-chronic approach relies on a complex 3-way interaction ('region X disease X task'), while the basal approach relied on simpler 'region X disease' to identify which hippocampal subregion is differentially targeted by disease. **Spatial resolution:** In the acute-on-chronic approach, activation tasks typically require very high temporal resolution, which, by necessity, compromises spatial resolution. The red pixels shown are those that are activated by a task (viewing novel faces [12]), and illustrate how it is difficult to determine in which hippocampal subregion they reside. In the basal approach, images can be acquired more slowly, thereby increasing spatial resolution, nearly by an order of magnitude [26]. Note, the upper red pixel in this basal hippocampal map of CBV (cerebral blood volume) is clearly within the dentate gyrus, while the lower red pixel is clearly within the CA1 subfield. **The 'n' factor**: This factor, reflecting the relationship between changes in oxygen metabolism and changes in CBF (cerebral blood flow), underlies the amplitude of fMRI signal. For acute changes in metabolism (indicated by the arrow in x-axis), 'n' varies from region to region and is affected by disease (indicated by the red and blue lines). Thus, identical shifts in neuronal metabolism will result in very different fMRI signal (see Fig. 4 for examples). 'n' is fixed and invariable for slower changes in metabolism, for example those that are induced by a disease. **The 'M' factor**: This factor, reflecting basal neurovascular properties such as deoxyhemoglobin and CBV, acts as an amplifier of BOLD during acute stimulus-induced changes in metabolism. Affected by disease, variations in 'M' will confound the BOLD response, confounding the interpretation when differences in stimulus-induced BOLD responses are observed in patients (see Fig. 4 for examples). In the basal approach, basal neurovascular properties are not confounds but the actual information used to pinpoint dysfunction.

firmed by numerous subsequent studies [21] the amplitude of the BOLD response signal are confounded by 'M'. Specifically, basal levels of dHB act as an 'amplifier' of the BOLD response, according to: $\Delta\, BOLD = M[1-(\frac{CBF\_t}{CBF\_0})^{\alpha-\beta}(\frac{CMRO2\_t}{CMRO2\_0})^{\beta}]$, where M is the proportionality constant that reflects basal levels of dHB, $\alpha$ is the relationship between CBV and CBF, and $\beta$ describes the oxygenation and field strength dependence of the BOLD effect. In principle, therefore, 2 stimuli that evoke identical changes in brain function will typically lead to very different BOLD responses (Fig. 4).

The final limitation of BOLD is the fact that it is inherently a relative, not quantitative, measure of changes in brain function. Although not necessarily a problem when using BOLD fMRI descriptively, when mapping cognitive processes for example, this becomes more problematic when trying to use fMRI for diagnostic purposes or for mapping changes over time. In these clinical scenarios a quantifiable, not relative, measure is preferable for obvious reasons.

## 4. The Basal approach

It should be pointed that many of the limitations described above can be, at least partially, addressed. For example, because CBF and CBV are quantitative measures, using them in an fMRI study, instead of relying on dHB, will address the 4th limitation. Additionally, CBF and CBV do not suffer from the vagaries associated with variability in 'M'. Nevertheless, mapping basal differences in fMRI, without an overt external stimulus, has a number of advantages that can address the limitations associated with the 'acute-on-chronic' experimental paradigm highlighted in the previous section (Fig. 3).

First, by comparing basal state maps of the hippocampal formation between patients and controls, disease-associated changes are driven by the disease itself, and are not biased by external sensory stimulation. Second, without needing to capture the effects of transient external stimulation, basal state maps can be generated more slowly, with nearly an order of magnitude higher spatial resolution [26]. Third, studies sug-

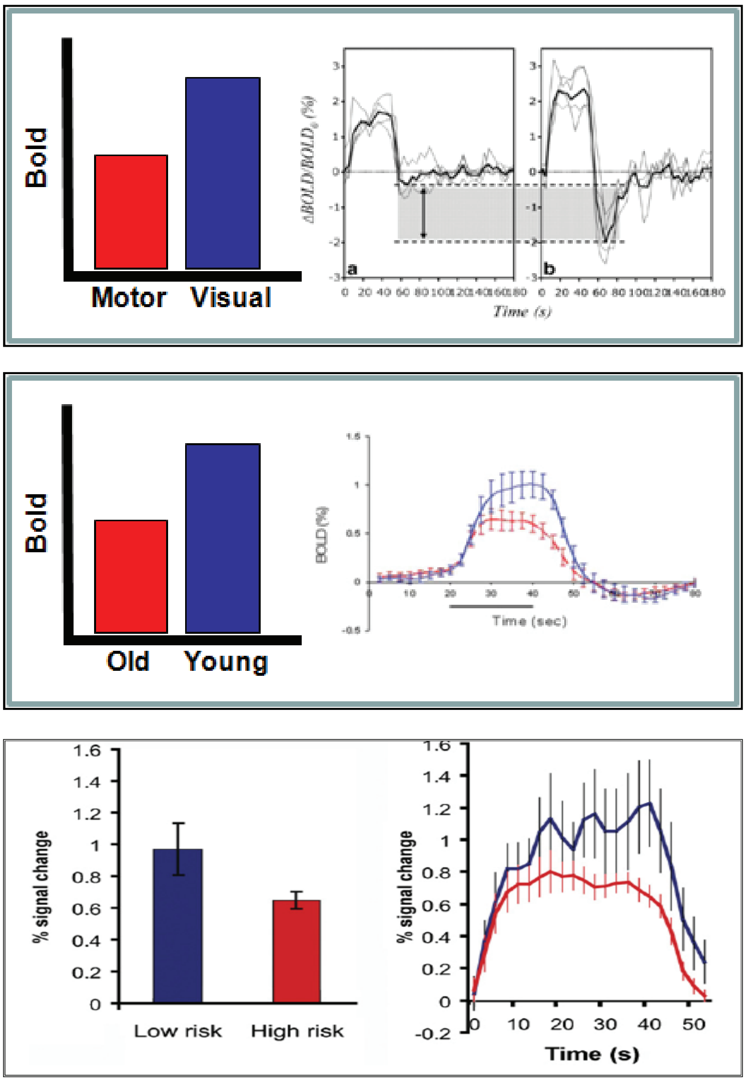

Fig. 4. Examples of how acute-on-chronic approach can lead to wrong interpretations. As described in the text, observed differences in the amplitude of stimulus-evoked BOLD (blood oxygen level dependent) response do not necessarily reflect underlying differences in neural activity, but rather, can reflect between-region or between-group neurovascular confounds (see Fig. 3). **Example 1** (upper panel): As reported [21], the larger stimulus-evoked BOLD response observed in the visual cortex compared to motor cortex (upper panel), does not reflect greater neural activity, but rather between-region differences in neurovascular coupling (the 'n' factor). **Example 2** (middle panel): As reported [23], the smaller BOLD response observed in the visual cortex of older compared to younger subjects does not reflect lower neural activity, but rather between-group differences in basal neurovascular properties (the 'M' factor). **Example 3** (lower panel): As reported [25], the smaller BOLD response observed in the hippocampus of APOE4 carriers ('high-risk') compared to non-carriers ('low-risk') does not reflect lower neural activity, but rather between-group differences in basal neurovascular properties (the 'M' factor).

gest that 'n' (the relationship between percent changes in CBF to percent changes in CMRO2) is fixed and invariant in the basal state [27], and is less affected by disease [28]. Fourth, in the basal approach 'M" is not a confound, but rather is the actual neurovascular information used to pinpoint dysfunction.

Thus, for the specific goal of pinpointing AD-associated hippocampal dysfunction, if dysfunction can be detected using the basal approach, it has, on balance, many advantages over the acute-on-chronic approach. The acute-on-chronic approach is recommended, however, if synaptic loss is too subtle to be detected by the basal approach; or, if the question is not pinpointing dysfunction, but rather asking how sensory representation and other cognitive processes are affected in AD.

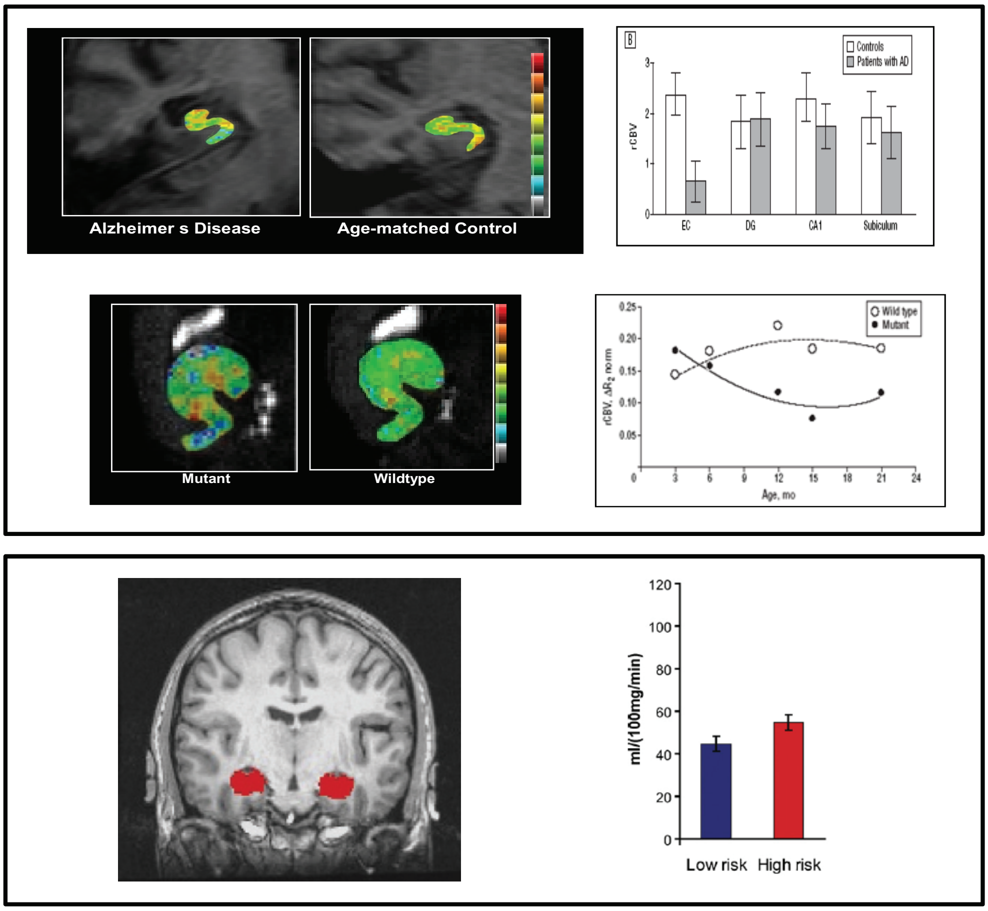

Fig. 5. Applying the basal approach in Alzheimer’s disease and disease risk factors. Basal mapping of CBV (cerebral blood volume) in human patients and a mouse model of disease (upper panel [26]) have documented that the entorhinal cortex is differentially vulnerable to disease, while the dentate gyrus is differentially vulnerable to aging. Basal mapping of CBF (cerebral blood flow) in the hippocampal formation (lower panel [25]) shows that APOE4 carriers (‘high risk’ for developing Alzheimer’s disease) have higher metabolism compared to APOE4 non-carriers.

## 5. Examples of recent findings using basal fMRI

Informed, in part, by the considerations discussed in the previous section, a number of recent studies have successfully used the basal fMRI to investigate AD and AD risk factors. For example, by applying a high-resolution variant of CBV to AD patients and mouse models of AD, Moreno et al. have shown that among the hippocampal subregions, the entorhinal cortex is found to have the greatest defects [26]. Importantly, because mice models develop synaptic loss but very little cell death, this study confirmed that fMRI is, in principle, sensitive to this early pathophysiological event. Because patients are presumed to have cell loss, even during mild cognitive impairment, arriving at this conclusion is impossible by relying on human studies alone. Furthermore, because mice models develop A$\beta$-related neurotoxicity, but do not develop tau pathology, this study suggests that abnormal accumulations of A$\beta$ are sufficient to cause entorhinal cortex dysfunction. Finally, by imaging mice harboring a disease-causing gene and their wildtype littermates across the life span, this study confirmed previous observations, suggesting that in contrast to AD, normal aging targets a different hippocampal subregion– the dentate gyrus.

A study by Fleisher et al. used basal CBF to examine the effect of the apolipoprotein E $\varepsilon$4 (APOE4) allele, the dominant genetic risk factor for late onset AD, on the hippocampal formation [25]. This study showed that compared to non-APOE4 carriers, APOE4 carriers have abnormal elevations of basal CBF, indicative of hypermetabolism. This is an important finding because recent studies suggest that increased neuronal metabolism causes an increase in A$\beta$ production. Thus, the finding by Fleisher et al. suggest a mechanism for how APOE4 can increase the risk for developing AD.

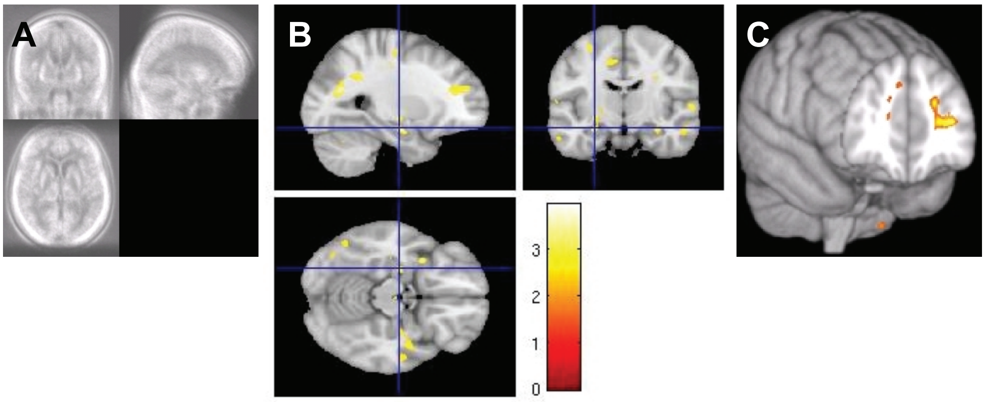

Fig. 6. Whole brain, voxelwise analysis of basal state fMRI. A) Mean CBV image of a large cohort ($n = 108$) of elderly, neurologically healthy, community-dwelling individuals. B) Voxelwise analysis comparing individuals with Mild Cognitive Impairment ($n = 38$) with elderly controls ($n = 108$), demonstrating areas of decreased CBV. C) Contrast map of age-associated differences in CBV.

## 6. Basal state fMRI to monitor disease course and treatment efficacy

In many ways, the use of basal state fMRI is ideal for tracking disease and treatment efficacy. As noted, the higher spatial resolution gives tremendous advantage in pinpointing small pathogenic sources of disease. Higher spatial resolution also increases the reliability of analysis of larger regions or networks of coordinated activity throughout the cortex and subcortex, increasing the likelihood of detecting subtle disease or age-related changes in cerebral function. For example, CBV can be computed across the entire brain and analyses can be conducted on a voxelwise basis. In the case of AD, once synaptic failure has progressed beyond the hippocampus, metabolic dysfunction occurs throughout cortical areas. Figure 6 shows a mean CBV image of normal healthy elderly drawn from a community sample and an example of the types of voxelwise analyses that can be conducted. As basal state fMRI approaches, such as CBV and CBF mapping, are unbiased by external stimuli and/or individual differences in performance on tasks, they are ideally suited for longitudinal studies in which random or disease-related fluctuations in task performance are intrinsically controlled.

## 7. Summary

Although there are a number of experimental fMRI approaches, using fMRI to map AD-associated changes in basal metabolism, a correlate of synaptic loss, is well-suited for the specific goal of detecting early AD-associated dysfunction. Applying this approach to human patients [26], aging non-human primates [29], mouse models of disease [26], and aging wildtype mice [26], has established a double anatomical dissociation that distinguishes the early stages of AD from normal cognitive aging. Specifically, whereas AD has been found to target the entorhinal cortex and spare the dentate gyrus, normal aging preferentially targets the dentate gyrus sparing the entorhinal cortex. This anatomical dissociation and the ability to visualize it in living patients can be used for detecting the early stages of AD, when it cognitively overlaps with normal aging, for monitoring the efficacy of therapeutic interventions [30], and for uncovering pathogenic mechanism in AD [31] and cognitive aging [32].

## References

[1] J.L. Whitwell, S.A. Przybelski, S.D. Weigand et al., 3D maps from multiple MRI illustrate changing atrophy patterns as subjects progress from mild cognitive impairment to Alzheimer's disease, *Brain* **130** (2007), 1777–1786.

[2] D.M. Jacobs, M. Sano, G. Dooneief, K. Marder, K.L. Bell and Y. Stern, Neuropsychological detection and characterization of preclinical Alzheimer's disease [comment] [see comments], *Neurology* **45** (1995), 957–962.

[3] E. Masliah, M. Mallory, L. Hansen, R. DeTeresa, M. Alford and R. Terry, Synaptic and neuritic alterations during the progression of Alzheimer's disease, *Neurosci Lett* **174** (1994), 67–72.

[4] L. Mucke, E. Masliah, G.Q. Yu et al., High-level neuronal expression of abeta 1-42 in wild-type human amyloid protein precursor transgenic mice: synaptotoxicity without plaque formation, *J Neurosci* **20** (2000), 4050–4058.

[5] D.J. Selkoe, Alzheimer's disease is a synaptic failure, *Science* **298** (2002), 789–791.

[6] S.A. Small, K. Kent, A. Pierce et al., Model-guided microarray implicates the retromer complex in Alzheimer's disease, *Ann Neurol* **58** (2005), 909–919.

[7] S.A. Small, Measuring correlates of brain metabolism with high-resolution MRI: a promising approach for diagnosing Alzheimer disease and mapping its course, *Alzheimer Dis Assoc Disord* **17** (2003), 154–161.

[8] J.W. Belliveau, D.N. Kennedy, Jr., R.C. McKinstry et al., Functional mapping of the human visual cortex by magnetic resonance imaging, *Science* **254** (1991), 716–719.

[9] K.K. Kwong, J.W. Belliveau, D.A. Chesler et al., Dynamic magnetic resonance imaging of human brain activity during primary sensory stimulation, *Proc Natl Acad Sci U S A* **89** (1992), 5675–5679.

[10] S. Ogawa, T.M. Lee, A.S. Nayak and P. Glynn, Oxygenation-sensitive contrast in magnetic resonance image of rodent brain at high magnetic fields, *Magn Reson Med* **14** (1990), 68–78.

[11] K. Uludag, D.J. Dubowitz, E.J. Yoder, K. Restom, T.T. Liu and R.B. Buxton, Coupling of cerebral blood flow and oxygen consumption during physiological activation and deactivation measured with fMRI, *Neuroimage* **23** (2004), 148–155.

[12] S.A. Small, G.M. Perera, R. DeLaPaz, R. Mayeux and Y. Stern, Differential regional dysfunction of the hippocampal formation among elderly with memory decline and Alzheimer's disease, *Ann Neurol* **45** (1999), 466–472.

[13] R.A. Sperling, B. Dickerson, J.F. Bates et al., fMRI studies of associative encoding in young and elderly controls and mild Alzheimer's disease, *J Neurol Neurosurg Psychiatry* **74** (2003), 44–50.

[14] S.A. Rombouts, F. Barkhof, D.J. Veltman et al., Functional MR imaging in Alzheimer's disease during memory encoding, *AJNR Am J Neuroradiol* **21** (2000), 1869–1875.

[15] R. Sperling, *Functional MRI Studies in Early Alzheimers Disease*, In: International Conference on Alzheimer's disease and Related Disorders; 2002; Stocholm, Sweden, 2002.

[16] D.G. Amaral and R. Insausti, The Hippocampal formation, in: *The Human Nervous System*, R. Paxinos, ed., San Diego: Academic Press, 1990.

[17] X. Zhao, E.S. Lein, A. He, S.C. Smith, C. Aston and F.H. Gage, Transcriptional profiling reveals strict boundaries between hippocampal subregions, *J Comp Neurol* **441** (2001), 187–196.

[18] S.A. Small, Age-related memory decline; current concepts and future directions, *Archives of Neurology* **58** (2001), 360–364.

[19] A. Bakker, C.B. Kirwan, M. Miller and C.E. Stark, Pattern separation in the human hippocampal CA3 and dentate gyrus, *Science* **319** (2008), 1640–1642.

[20] O. Leontiev and R.B. Buxton, Reproducibility of BOLD, perfusion, and CMRO2 measurements with calibrated-BOLD fMRI, *Neuroimage* **35** (2007), 175–184.

[21] P.A. Chiarelli, D.P. Bulte, D. Gallichan, S.K. Piechnik, R. Wise and P. Jezzard, Flow-metabolism coupling in human visual, motor, and supplementary motor areas assessed by magnetic resonance imaging, *Magn Reson Med* **57** (2007), 538–547.

[22] B.M. Ances, O. Leontiev, J.E. Perthen, C. Liang, A.E. Lansing and R.B. Buxton, Regional differences in the coupling of cerebral blood flow and oxygen metabolism changes in response to activation: implications for BOLD-fMRI, *Neuroimage* **39** (2008), 1510–1521.

[23] B.M. Ances, C.L. Liang, O. Leontiev et al., Effects of aging on cerebral blood flow, oxygen metabolism, and blood oxygenation level dependent responses to visual stimulation, *Hum Brain Mapp* **30** (2009), 1120–1132.

[24] T.L. Davis, K.K. Kwong, R.M. Weisskoff and B.R. Rosen, Calibrated functional MRI: mapping the dynamics of oxidative metabolism, *Proc Natl Acad Sci U S A* **95** (1998), 1834–1839.

[25] A.S. Fleisher, K.M. Podraza, K.J. Bangen et al., Cerebral perfusion and oxygenation differences in Alzheimer's disease risk, *Neurobiol Aging* **4** (2008), 4.

[26] H. Moreno, W.E. Wu, T. Lee et al., Imaging the abeta-related neurotoxicity of Alzheimer disease, *Arch Neurol* **64** (2007), 1467–1477.

[27] P.T. Fox and M.E. Raichle, Focal physiological uncoupling of cerebral blood flow and oxidative metabolism during somatosensory stimulation in human subjects, *Proc Natl Acad Sci U S A* **83** (1986), 1140–1144.

[28] H. Fukuyama, M. Ogawa, H. Yamauchi et al., Altered cerebral energy metabolism in Alzheimer's disease: a PET study, *J Nucl Med* **35** (1994), 1–6.

[29] S.A. Small, M.K. Chawla, M. Buonocore, P.R. Rapp and C.A. Barnes, From The Cover: Imaging correlates of brain function in monkeys and rats isolates a hippocampal subregion differentially vulnerable to aging, *Proc Natl Acad Sci U S A* **101** (2004), 7181–7186.

[30] A.C. Pereira, D.E. Huddleston, A.M. Brickman et al., An *in vivo* correlate of exercise-induced neurogenesis in the adult dentate gyrus, *Proc Natl Acad Sci U S A* **104** (2007), 5638–5643.

[31] S.A. Small, Retromer sorting: a pathogenic pathway in late-onset Alzheimer disease, *Arch Neurol* **65** (2008), 323–328.

[32] E. Wu, A. Brickman, J. Luchsinger et al., The brain in the age of old: The hippocampal formation is differentially affected by diseases of late-life, *Annals of Neurology* (2008), in press.
